# Probiotic-derived ferrichrome induces DDIT3-mediated antitumor effects in esophageal cancer cells

**DOI:** 10.1016/j.heliyon.2024.e28070

**Published:** 2024-03-15

**Authors:** Takehito Kunogi, Hiroaki Konishi, Aki Sakatani, Kentaro Moriichi, Chikage Yamamura, Koji Yamamoto, Shin Kashima, Katsuyoshi Ando, Nobuhiro Ueno, Hiroki Tanaka, Toshikatsu Okumura, Mikihiro Fujiya

**Affiliations:** a Division of Gastroenterology, Department of Internal Medicine, Asahikawa Medical University, Asahikawa, 078-8510, Japan; b Department of Gastroenterology and Advanced Medical Sciences, Asahikawa Medical University, Asahikawa, 078-8510, Japan; c Division of Tumor Pathology, Department of Pathology, Asahikawa Medical University, Asahikawa, 078-8510, Japan

**Keywords:** Esophageal cancer, Probiotics, Ferrichrome, Apoptosis, DDIT3

## Abstract

Esophageal cancer, which is common among the elderly, has the poorest prognosis among gastrointestinal cancers. Previously, we demonstrated that ferrichrome, produced by the probiotic *Lactobacillus casei*, exhibited anti-tumor effects in various gastrointestinal cancers, including colorectal and gastric cancers, with minimal effects on non-cancerous intestinal cells. However, it remains unclear whether ferrichrome exerts anti-tumor effects in esophageal cancer. A sulforhodamine B assay revealed that ferrichrome suppressed esophageal adenocarcinoma (OE33, OE19) and squamous cell carcinoma (KYSE70) cells. Ki-67 staining indicated that ferrichrome inhibited the proliferation of esophageal cancer cells. Cell cycle analysis showed that ferrichrome inhibited the DNA synthesis. TUNEL staining revealed that ferrichrome-induced DNA fragmentation. We also confirmed the cleavage of caspase-9 and PARP in ferrichrome-treated cells. Reverse transcription polymerase chain reaction demonstrated an increase in the mRNA of DNA damage-inducible transcript 3 (DDIT-3), a key regulator of programmed cell death, in ferrichrome-treated OE33 cells. In an *in vivo* OE33 xenograft model, intraperitoneal administration of 5-mg/kg ferrichrome for 14 days resulted in an almost complete inhibition of tumor growth. However, 14 days of intraperitoneal administration of 20-mg/kg 5-fluorouracil (5-FU), but not 20-mg/kg ferrichrome, induced weight loss and myelosuppression in both young and aged mice. Our findings indicate that ferrichrome induces DNA damage-inducible transcript-3, thereby producing anti-tumor effects, including cell cycle arrest and apoptosis, with minimal adverse effects in esophageal cancer cells. This illustrates the high potential of ferrichrome as an anti-tumor drug against esophageal carcinoma.

## Highlights

• Probiotic-derived ferrichrome has anti-tumor effects against esophageal cancer.
• Apoptosis is induced by ferrichrome-mediating DDIT3 in esophageal carcinoma.
• Ferrichrome has a greater antitumor effect and a safer profile than 5-FU.

## 1 Introduction

Esophageal cancer ranks as the sixth leading cause of death among all cancers worldwide, and, among the gastrointestinal cancers, it exhibits particularly poor prognoses [1]. Although there have been advancements in treatment modalities, including endoscopic and surgical interventions as well as chemotherapy, and the emergence of immune checkpoint inhibitors [2,3], the 5-year survival rate for advanced esophageal cancer remains extremely low at <20% [2]. Notably, esophageal cancer frequently affects elderly individuals, with a median age of diagnosis of 65 and 68 years for men and women, respectively [4]. However, conventional chemotherapy regimens, including cisplatin–5-fluorouracil (5-FU) combination therapy, often pose challenges for elderly patients because of the frequent occurrence of adverse effects, including myelosuppression and gastrointestinal disorders [5]. Consequently, there is a pressing need to develop new therapeutic agents that not only exhibit superior cytotoxicity against cancer cells but also minimize adverse effects, especially in the elderly population.

Probiotics, known for their beneficial effects on host mammals, have the potential to reduce the occurrence of colon adenomas [6]. Therefore, in the field of gastrointestinal cancer therapeutics, probiotic administration has been explored as a strategy [7,8]. Although probiotics are expected to be applied as safe antitumor agents, the antitumor abilities of live bacteria have been controversial in clinical studies [6,9], as the antitumor activity of live probiotics can be influenced by various factors, including individual gut conditions and dysbiosis resulting from factors such as diet, lifestyle, diseases, and chemotherapy.

In the past decade, several probiotic-derived antitumor molecules have been identified and expected to be candidates of cancer therapeutic drugs with safety profiles. For instance, Sakatani et al. demonstrated that long-chain polyphosphates derived from *Lactobacillus brevis*, identified as intestinal cytoprotective molecules, inhibit the growth of colon cancer cells by activating the ERK pathway [10]. Tsai et al. also reported that M2163 and M2386, produced by *Lactobacillus casei*, exert their antitumor effects by inducing apoptosis in colon cancer cells [11].

We also identified ferrichrome as an antitumor molecule in the culture supernatant of *L. casei* [12]. Ferrichrome is a type of siderophore, which is a small, high-affinity molecule produced by microorganisms for iron chelation [[13], [14], [15]]. Ferrichrome has antitumor potential in colon cancer cells by inducing the expression of DNA damage-inducible transcript-3 (DDIT3), surpassing the efficacy of conventional anticancer drugs such as 5-FU and cisplatin [16]. It also exhibited anti-tumor effects on gastric and pancreatic cancer cells [16,17]. Recently, Chaib et al. highlighted the potential of combination therapy involving ferrichrome and immune checkpoint inhibitors, such as PD-L1 inhibitors, in pancreatic cancer treatment [19]. However, it remains unclear whether ferrichrome exerts anti-tumor effects in refractory esophageal cancer. It is worth noting that the intravenous injection of ferrichrome, at doses effective for its anticancer activity, did not induce adverse events including myelosuppression, kidney failure, or liver failure, which are common side effects associated with conventional chemotherapy [18]. Therefore, there is a need for the clinical application of ferrichrome as an anticancer drug, particularly for high-risk patients, such as the elderly and those with primary diseases. Chemotherapy poses distinct challenges for elderly patients because of their age-related metabolic changes, which can result in the development of more severe side effects as compared to younger patients. Thus, it is imperative to evaluate the safety of ferrichrome, specifically among elderly patients, considering their unique vulnerabilities and the drug's potential interactions with existing health conditions.

The present study aimed to examine the potential anti-tumor effects of ferrichrome on esophageal cancer cells and to assess the safety of ferrichrome, specifically in an elderly population, using an aged mouse model.

## 2 Materials and methods

### 2.1 Cell culture

Human esophageal adenocarcinoma cell lines, namely OE19 (EC96071721-G0, CVCL_1622) and OE33 (EC96070808-F0, CVCL_0471), as well as the human esophageal squamous cell carcinoma cell line, KYSE70 (EC94072012-F0, CVCL_1356), were procured from KAC, Inc (Japan). These cell lines were cultivated in the RPMI-1640 medium (FUJIFILM Wako Pure Chemical Co. Ltd., Japan), which was supplemented with 10% (vol/vol) fetal bovine serum, 50-U/mL penicillin, and 50-mg/mL streptomycin (Thermo Fisher Scientific, USA).

### 2.2 Ferrichrome

Ferrichrome was purchased from Merck KGaA, Darmstadt, Germany, and was prepared as a stock solution by dissolving it in distilled water at a concentration of 5 mg/mL. To achieve the desired test concentrations, ferrichrome was further diluted using DMEM high glucose medium before its administration to the cells. Subsequent assays were performed following this treatment.

### 2.3 Sulforhodamine B (SRB) assay

Cells were initially seeded at a density of 1–2 × 10^4^ in 96-well microplates. After an incubation period of 24 h, the cells were treated with ferrichrome. Subsequently, at time intervals of 24, 72, and 120 h, the cells were treated with a solution containing 5% (w/v) trichloroacetic acid in distilled water. Then, the cells were washed four times with distilled water, air-dried at room temperature, and stained with a 0.057% solution of sulforhodamine B (SRB) in 0.1% (v/v) acetic acid in distilled water for 30 min. Following the staining process, the microplate was washed four times with a solution of 0.1% acetic acid and allowed to air-dry at room temperature. The stained cells were subsequently dissolved in 10-mM Tris buffer, and their optical density was measured at a wavelength of 510 nm.

### 2.4 Immunocytochemistry

Cells were initially seeded at a density of 1 × 10^5^ in 4-well plastic chamber slides. After a 24-h incubation period, the cells were treated with ferrichrome. Subsequently, at the 72-h mark, the cells were fixed using a 4% paraformaldehyde solution for 1 h at room temperature. Following fixation, they were washed with PBS and then permeabilized with a 0.2% Triton-X/PBS solution for 30 min. After permeabilization, the cells underwent blocking using the SuperBlock™ (PBS) Blocking Buffer from Thermofisher Scientific for 1 h. Next, the cells were treated with a Ki67 antibody (Novus Biologicals, LLC., USA) in SuperBlock™ (PBS) Blocking Buffer and incubated overnight at 4 °C. Following thorough washing with PBS, the cells were exposed to Alexa Fluor 488 (Thermo Fisher Scientific) as a secondary antibody in SuperBlock™ (PBS) Blocking Buffer for 1 h at room temperature. Further PBS washing was conducted, and the cells were treated with Hoechst 33,342 (ThermoFisher Scientific) diluted in PBS for 3 min, followed by another PBS wash. Finally, the cells were sealed with an anti-fade mounting medium, VectamountAQ (VECTOR Laboratories), and observed using a fluorescence microscope.

### 2.5 TUNEL staining

Cells were initially seeded at a density of 1 × 10^5^ in 4-well plastic chamber slides. After 24 h, the cells were treated with ferrichrome. At the 72-h time point, the cells were fixed using a 4% paraformaldehyde solution for 1 h at room temperature and subsequently subjected to thorough washing with PBS. The slides were stained using an In Situ Cell Death Detection Kit with TMR red (Roche Diagnostics, IN, USA) according to the manufacturer's instructions. After staining, the slides were washed again with PBS, sealed with VectamountAQ (VECTOR Laboratories), and visualized under a fluorescence microscope (KEYENCE Corporation, Osaka, Japan) to identify TUNEL-positive cells.

### 2.6 Flow cytometry

We initially seeded the cells at a density of 0.375 × 10^6^ in 60-mm dishes. After a 24-h incubation, the cells were treated with ferrichrome. At the 72-h time point, we conducted the following procedures. First, we collected the culture supernatant. Subsequently, we added 2.5 mL of PBS to the dishes and transferred it to a separate tube. Next, we applied trypsin to the dishes and harvested the cells by adding RPMI-1640 supplemented with 10% fetal bovine serum to the tube. After centrifugation at 2000 rpm for 5 min, the cells were suspended in 2 mL of PBS. To fix the cells, we added 4 mL of 100% ethanol and incubated them with 25 U/mL RNase (Wako Pure Chemicals, Osaka, Japan) at room temperature for 20 min. We then introduced a propidium iodide solution at a final concentration of 50 μg/mL. The samples were subsequently analyzed by flow cytometry, with 20,000 events recorded for each sample. Data analysis was performed using Cell Quest Pro (BD Bioscience) and ModFit LT (BD Bioscience).

#### 2.6.1 RT-PCR

The cells were initially seeded at a density of 2 × 10^5^ in six-well dishes. After a 24-h incubation, the cells were treated with ferrichrome. Subsequently, the cells were washed with PBS, and RNA extraction was performed using the RNAeasy Mini Kit (Qiagen). The mRNA was then reverse transcribed using the cDNA Reverse Transcription Kit from ThermoFisher Scientific. Following reverse transcription, cDNA amplification was conducted using primers specific for DDIT3 (ThermoFisher Scientific). Gene expression signals were detected using the Applied Biosystems 7300 RT-PCR system. To determine the average mRNA expression levels, the 18S ribosomal RNA (ThermoFisher Scientific) expression levels were used as a reference.

### 2.7 Western blotting

The cells were washed with PBS and subsequently extracted using NP-40 Cell Lysis Buffer (ThermoFisher Scientific) containing Complete Protease Inhibitor (Sigma Aldrich) and Halt Phosphatase Inhibitor Cocktail (ThermoFisher Scientific). Equal amounts of protein were separated using sodium dodecyl sulfate-polyacrylamide gel electrophoresis with 12.5% gel. After electrophoresis, the proteins were transferred onto a nitrocellulose membrane and blocked using SuperBlock™ (PBS) Blocking Buffer from ThermoFisher Scientific. Following blocking, the blots were incubated overnight at 4 °C with primary antibodies. Afterward, the blots were then washed with PBS, incubated with secondary antibodies for 1 h, washed again with PBS, and detected using a Luminograph. The protein levels were normalized to actin expression using antibodies from BD Transduction Laboratories, Lexington, KY, USA.

### 2.8 Statistical analysis

The assay data from the two groups were analyzed using Student's unpaired *t*-test. For the assay data involving more than three groups, an analysis of variance was performed, followed by Tukey's post hoc test. Statistical significance was defined as P < 0.05.

## 3 Results

### 3.1 Ferrichrome inhibited the growth of esophageal cancer cells

**Fig. 1:**
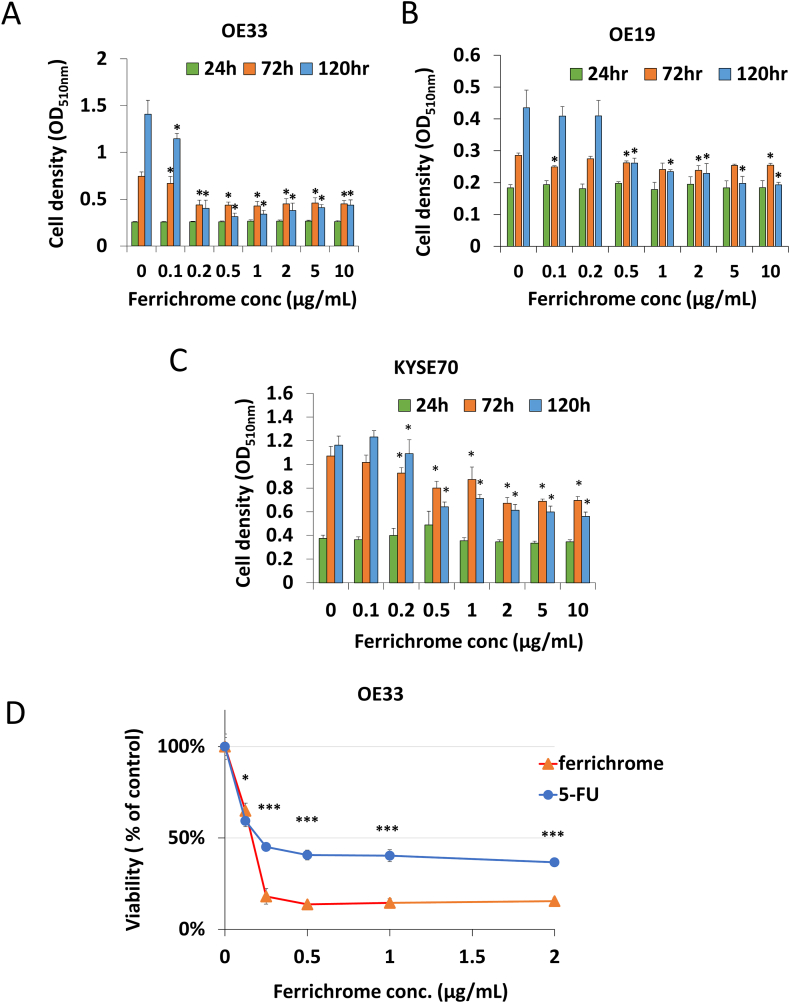
Ferrichrome inhibited the growth of esophageal adenocarcinoma cells. The results of the SRB assay indicate a significant decrease in the number of esophageal cancer cells treated with ferrichrome in a concentration-dependent manner (OE33; n = 6, OE19; n = 3, KYSE-70; n = 5) when compared to the control group (A–C). To further evaluate the tumor-suppressive effects of ferrichrome and compare them with those of 5-FU, we treated the OE33 cells with either ferrichrome or 5-FU. Notably, ferrichrome demonstrated a more potent tumor-suppressive effect as compared to 5-FU in OE33 cells at the 120-h time point (n = 5) (D). Error bars and numbers represent standard deviation (SD), and each value was statistically compared between the ferrichrome and 5-FU groups. Significance levels are denoted as follows: *p < 0.05, ***p < 0.001 by Student's t-test.

To investigate the tumor-suppressive effect of ferrichrome on esophageal cancer cells *in vitro*, we introduced ferrichrome to the esophageal cancer cell lines OE19, OE33, and KYSE70. Ferrichrome demonstrated concentration-dependent inhibition of growth in both the esophageal adenocarcinoma (OE19 and OE33) and squamous carcinoma (KYSE70) cell lines (Fig. 1A–C). For a comparative assessment of the tumor-suppressive effects of ferrichrome and 5-FU, we treated the OE33 cells with either ferrichrome or 5-FU. Remarkably, ferrichrome exhibited a more potent tumor-suppressive effect as compared to 5-FU (Fig. 1D).

### 3.2 Ferrichrome inhibited cell proliferation and induced apoptosis in esophageal cancer cells

**Fig. 2:**
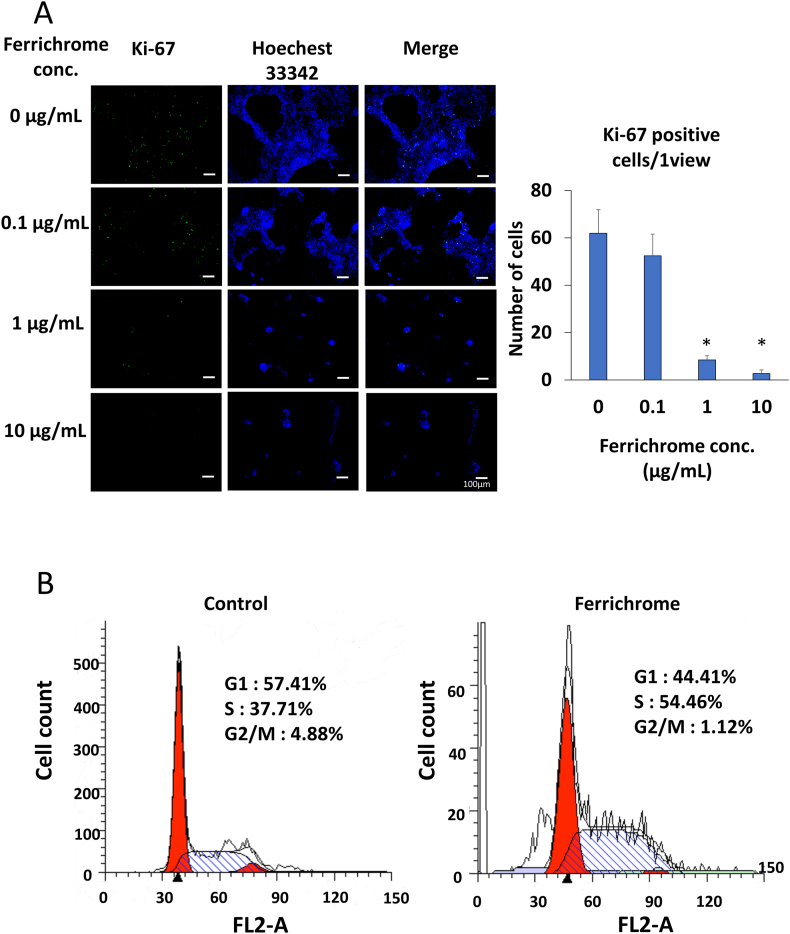
Ferrihcrome inhibited cell proliferation through the S-phase arrest in esophageal cancer cells Immunostaining of Ki-67 revealed a significant reduction in Ki-67-positive cells upon ferrichrome treatment in OE33 cells (n = 4) (A). Flow cytometry analysis demonstrated the accumulation of ferrichrome-treated cells in the S phase, indicating that DNA synthesis was inhibited by ferrichrome treatment in esophageal cancer cells (B). Error bars and numbers indicate the standard deviation (SD), and each value was statistically compared with that of the control group. Significance is denoted as *p < 0.05 by Student's t-test.

To assess the impact of ferrichrome treatment on cell proliferation, we performed immunocytochemistry by Ki-67 staining. The immunostaining results demonstrated a significant reduction in Ki-67-positive cells in response to ferrichrome treatment in a concentration-dependent manner in OE33 cells (Fig. 2A). To further analyze the effect of ferrichrome on the cell cycle of esophageal cancer cells, we performed a flow cytometry analysis (Fig. 2B), which revealed an accumulation of ferrichrome-treated cells in the S phase, indicating that ferrichrome treatment inhibited the DNA synthesis process in esophageal cancer cells.

**Fig. 3:**
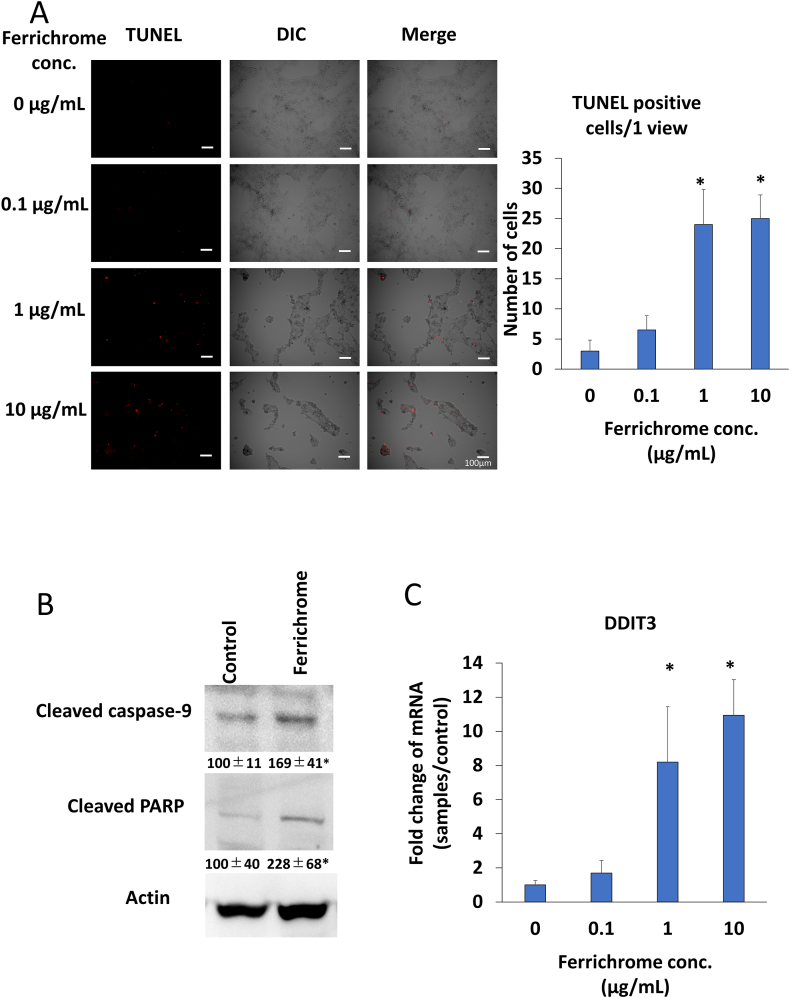
Ferrihcrome-induced apoptosis on esophageal cancer cells TUNEL staining clearly demonstrated an increase in the number of TUNEL-positive cells in the ferrichrome-treated group as compared with the control group (n = 4) (A). Ferrichrome treatment induced the fragmentation of caspase-9 and PARP at 72 h in OE33 cells (n = 3) (B). Additionally, the mRNA expression of DDIT3 was significantly upregulated in a concentration-dependent manner at 72 h in OE33 cells (C). Error bars and numbers represent the standard deviation (SD), and each value was statistically compared with that of the control. Significance is indicated as *p < 0.05 by Student's t-test.

Next, we conducted TUNEL staining to evaluate whether ferrichrome-induced programmed cell death in esophageal cancer cells. TUNEL staining results demonstrated a concentration-dependent increase in TUNEL-positive cells in response to ferrichrome treatment as compared with the control cells (Fig. 3A). Furthermore, Western blotting revealed that ferrichrome significantly induced caspase-9 and PARP cleavage (Fig. 3B), suggesting that ferrichrome-induced apoptosis in esophageal cancer cells.

### 3.3 Ferrichrome exhibited anti-tumor effects by inducing the expression of DDIT3 mRNAs

In our previous studies, we established that ferrichrome induces the expression of DDIT3, an apoptosis-associated transcription factor, thereby demonstrating its anti-tumor properties that promote apoptosis in colon and gastric cancer cells [11,15]. Therefore, we sought to determine whether ferrichrome could also induce DDIT3 expression in esophageal cancer cells. Our findings revealed a significant concentration-dependent increase in DDIT3 mRNA expression with ferrichrome treatment in OE33 cells (Fig. 3C). These results strongly indicate that ferrichrome induces expression of DDIT3, consequently exerting anti-tumor effects in esophageal cancer cells.

### 3.4 Ferrichrome inhibited tumor enlargement in a mouse xenograft model

**Fig. 4:**
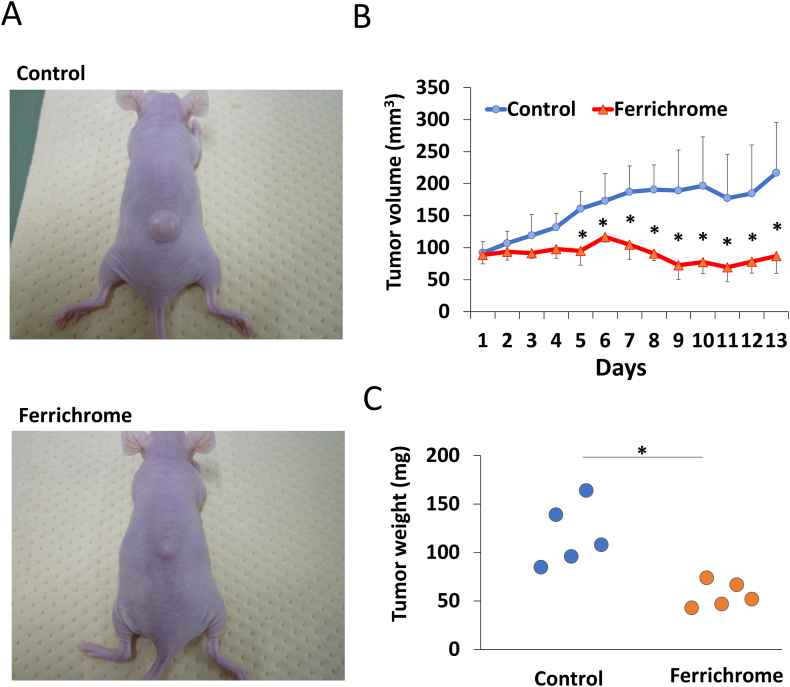
Ferrichrome exerts anti-tumor effects in an *in vivo* xenograft model of OE33 cells PBS and 100 μg of ferrichrome were intraperitoneally administered to the OE33-transplanted mice (A). Ferrichrome exhibited a significant suppression of tumor size in the treatment group as compared with the control group (n = 5) (B, C). Error bars and numbers represent the standard deviation (SD), and each value was statistically compared with the control. Significance is indicated as *p < 0.05 by Student's t-test.

To validate the antitumor effect of ferrichrome *in vivo*, we transplanted OE33 cells into nude mice and administered 5 mg/kg of ferrichrome via intraperitoneal injection daily (Fig. 4A). Remarkably, ferrichrome significantly inhibited the tumor growth starting from day 4 (Fig. 4B), and tumor weight in the ferrichrome-treated group was significantly reduced as compared with that of the control group (Fig. 4C). These findings strongly suggest that ferrichrome exhibits potent anti-tumor effects in esophageal cancer *in vivo*.

### 3.5 Ferrichrome showed low cytotoxicity in the mouse repeated dosing model

**Fig. 5:**
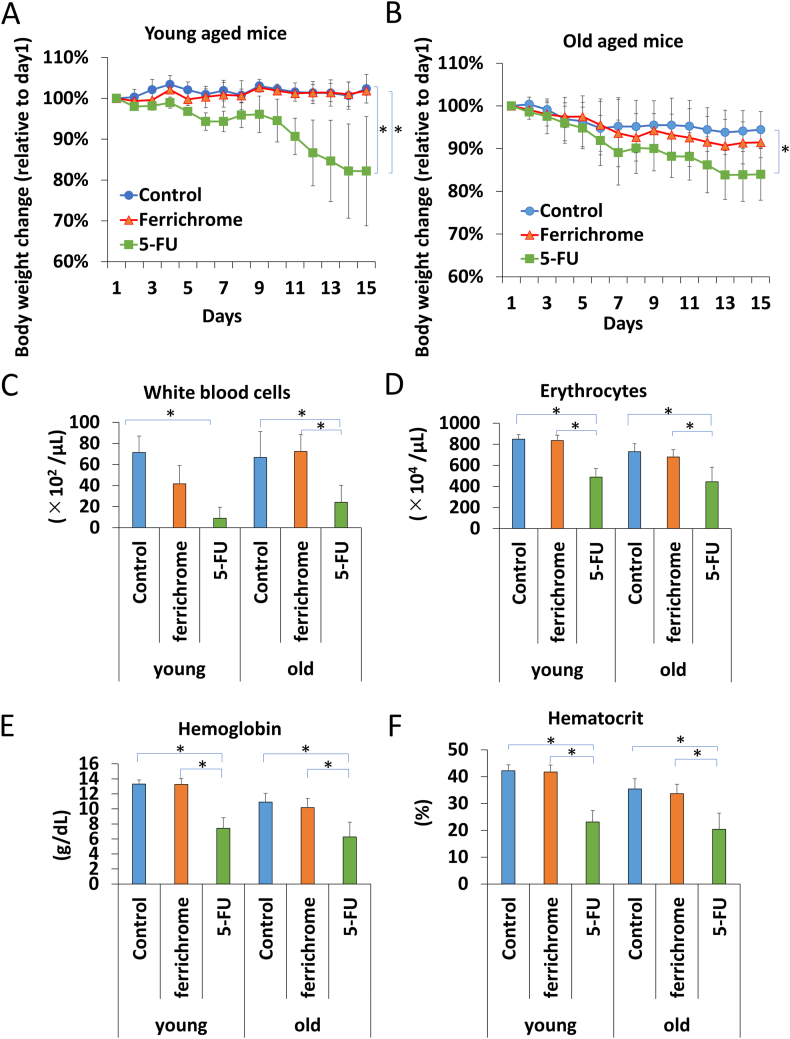
Ferrichrome did not induce hematopoietic abnormality in young- and old-aged mice The body weight of the 5-FU-treated group, but not the ferrichrome-treated group, showed a significant reduction in both the young-aged (A) and old-aged mice (n = 5) (B). Furthermore, the reduction in erythrocytes and white blood cells, as well as a decrease in hemoglobin and hematocrit levels, was observed in the 5-FU treated group, but not in the ferrichrome group (n = 5) (C–F). Error bars and numbers represent the standard deviation (SD), and each value was statistically compared among the control, ferrichrome, and 5-FU groups. Significance is indicated as *p < 0.05 by Student's t-test.

To assess the safety of ferrichrome for potential use in elderly patients, experiments were conducted using an aged mice model. We administered 20 mg/kg of ferrichrome, equivalent to four times the drug's effective dose, or 20 mg/kg of 5-FU for a duration of 14 days intraperitoneally and subsequently evaluated the safety parameters. Notably, no drug-induced mortality was observed in either group (data not shown). However, the body weight of the 5-FU-treated group exhibited significant losses in both young-aged (8–10 weeks old) (Fig. 5A) and old-aged (80–82 weeks old) mice (Fig. 5B) [[20], [21], [22]]. Contrarily, the ferrichrome-treated group did not show any significant loss in body weight as compared with the control group.

Furthermore, we observed symptoms of myelosuppression, including decreased erythrocyte counts, white blood cell counts, and hemoglobin and hematocrit levels in the 5-FU-treated mice. However, these myelosuppressive effects were not observed in either young- or old-aged mice treated with ferrichrome (Fig. 5C–F). These results indicate that the safety profile of ferrichrome is superior to that of 5-FU, regardless of the subjects’ age.

## 4 Discussion

The present study revealed the remarkable anti-tumor potential of ferrichrome, derived from probiotic bacteria, against esophageal cancer cells. Ferrichrome not only effectively reduced cell growth but also induced caspase-mediated apoptosis by upregulating DDIT3, an apoptosis-associated transcription factor. Importantly, this anti-tumor activity was confirmed in mice transplanted with esophageal cancer cells, with no cytotoxicity, including myelosuppression, observed in ferrichrome-treated mice. Notably, ferrichrome's anti-tumor effect was superior to that of the classical antitumor agent 5-FU against esophageal cancer cells. Consequently, ferrichrome holds promise as a valuable anti-tumor agent in esophageal cancer treatment.

The present study has revealed a novel mechanism underlying a bacteria-associated anti-tumor function in esophageal cancer. Previous research has linked dysbiosis in esophageal microbiota to the progression of esophageal cancer [23,24]. However, the direct antitumor impact of bacteria on tumor suppression had not been previously proposed. The present study clearly demonstrated for the first time that anti-tumor bacteria, such as *L. casei*, can directly inhibit the progression of esophageal cancer through the mediation of bacteria-derived anti-tumor molecules, without altering the esophageal microbiota.

DDIT3, a tumor suppressor protein, exerts its tumor-suppressive effects by inducing apoptosis through ER stress [25]. Our previous research had demonstrated the induction of DDIT3 by ferrichrome treatment in colorectal and gastric cancer cells. Similarly, in esophageal cancer cells, ferrichrome displayed an anti-tumor activity by promoting DDIT3-mediated apoptosis (Fig. 3C). These findings strongly suggest that the anti-tumor pathway involving DDIT3-mediated apoptosis is a common feature of gastrointestinal cancer cells. In clinical studies involving human specimens, low DDIT3 expression has been identified as a poor prognostic factor in gastrointestinal cancers, including gastric cancer [26]. Intriguingly, DDIT3 overexpression has been correlated with a reduced risk of breast cancer recurrence [27]. Furthermore, DDIT3 overexpression can promote esophageal cancer cell apoptosis [28,29]. These insights indicate that agents capable of inducing DDIT3, such as ferrichrome, could be applied therapeutically in various tumors exhibiting DDIT3 downregulation. Moreover, it is worth noting that a single-nucleotide polymorphism (SNP) has been reported in DDIT3 genes. Specifically, the missense SNP DDIT3-rs697221 has been reported to be associated with an increased risk of lung cancer [30]. Future investigations regarding the antitumor effects on tumors with DDIT3 gene function loss due to SNPs, genetic mutations, and other causes will provide further insights into the potential of DDIT3-upregulating agents, including ferrichrome, and the utility of DDIT3 as a prognostic factor.

Immunocytochemistry using Ki-67 and TUNEL staining clearly demonstrated that ferrichrome induced cell growth arrest and apoptosis in esophageal cancer cells. Although previous studies have shown that ferrichrome induces apoptosis in colon and gastric cancer cells, the mechanism by which ferrichrome inhibits cell growth in gastrointestinal cancer cells has remained unexplored [12,16]. The results of our flow cytometry analysis indicated that ferrichrome arrests the cell cycle in the S phase in esophageal cancer cells. Interestingly, we have previously observed the S-phase arrest by ferrichrome treatment in pancreatic cancer cells [17] but not in gastric cancer cells [15], suggesting that ferrichrome-induced cell cycle arrest may be dependent on the specific type of cancer cell. The dysfunction in DNA synthesis or repair can activate the ATM/ATR sensor, leading to the S-phase arrest [31]. Classical chemotherapeutic agents, such as 5-FU and cisplatin, inhibit DNA replication directly through intercalation, thereby inducing S-phase arrest [32]. Contrarily, the hydroxamate arms of ferrichrome are negatively charged; hence, it unlikely that they directly bind to the negatively charged DNAs and inhibit the DNA synthesis. Therefore, it is plausible that ferrichrome indirectly activates the ATM/ATR sensor through the stress-associated molecules, such as DDIT3, to inhibit DNA synthesis or repair mediated by DNA polymerase and topoisomerase, thereby inducing the S-phase arrest. The specific mechanisms underlying this process require further elucidation in future studies.

Patients with gastrointestinal cancer, especially esophageal cancer, are often elderly, making the need for safe chemotherapeutic agents particularly pressing. 5-FU is a commonly employed anti-tumor agent that, at times, leads to severe adverse events, including myelosuppression, frequently necessitating the discontinuation of chemotherapy [5]. Our drug safety assessment of ferrichrome, which was conducted on both young and aged mice, clearly demonstrated that ferrichrome did not result in weight loss or myelosuppression, even among the elderly mice. This strongly suggests that ferrichrome has a safer profile as compared to 5-FU. These findings underscore the attractiveness of ferrichrome as a candidate molecule for gastrointestinal cancer treatment, given its high anti-tumor efficacy and low risk of adverse events.

In the current chemotherapy regimens, employing a combination of several agents, such as FOLFOX, is a common practice. Preclinical studies are already underway to evaluate the potential of ferrichrome as a novel chemotherapeutic agent, including the assessment of its synergistic effects when combined with other chemotherapeutics, such as PD-L1 inhibitors [19]. We have clearly demonstrated that the anti-tumor properties of ferrichrome surpass those of 5-FU (Fig. 1D). Notably, the mechanism of action of ferrichrome differs from that of 5-FU, which primarily inhibits DNA synthesis. Consequently, we anticipate that ferrichrome will exhibit synergistic effects when combined with the current chemotherapeutic agents, including 5-FU. Moreover, considering that 5-FU often leads to adverse events, such as myelosuppression, ferrichrome offers a much safer alternative (Fig. 5). Reducing the reliance on traditional chemotherapeutics by incorporating ferrichrome into the treatment regimens could pave the way for the development of safer approaches to treating esophageal cancer in the future.

Our previous research has provided evidence of ferrichrome's anti-tumor effects in pancreatic cancer cells, mediated through the p53 signaling pathway [15]. Intriguingly, the esophageal cancer cell line OE33 carries a point mutation in exon 5 (c.404G > A, p. C135Y) within the coding region of the p53 gene. Owing to this mutation, OE33 cells have lost their tumor suppressive functions via p53 signaling, including the transcription of tumor suppressive mRNAs [33]. Remarkably, our SRB and xenograft assays clearly demonstrated that ferrichrome exerts tumor suppressive effects in OE33 cells. This suggests that ferrichrome's anti-tumor effects in esophageal cancer cells are mediated through a DNA damage-responsive pathway, rather than on the p53 tumor suppressive pathway, which is inactive in these cells.

In the present study, we assessed the anti-tumor efficacy of ferrichrome against both squamous cell carcinoma and adenocarcinoma. Our SRB assay results revealed that the anticancer effect of ferrichrome on esophageal adenocarcinoma, specifically OE33 cells, was more pronounced than that on esophageal squamous cell carcinoma, exemplified by KYSE-70 cells. Interestingly, our previous investigations have consistently shown that ferrichrome exhibits robust anti-tumor effects in adenocarcinoma cells, including colorectal (SW620) [12], pancreatic (SUIT-2) [18], and gastric (MKN-45) adenocarcinomas [16]. These findings collectively suggest that ferrichrome possesses potent cytotoxicity against gastrointestinal adenocarcinoma but demonstrates relatively weaker cytotoxicity against squamous cell carcinoma. To gain further insights, future studies should aim to determine whether this difference in cytotoxicity is specific to the particular squamous cell carcinoma cell line used in the present study or if ferrichrome indeed exhibits a greater potency against adenocarcinoma.

We presented the potential clinical applications of ferrichrome and its advantages in esophageal cancer cell therapy using *in vitro* and *in vivo* xenograft models and aged mouse models. Our study findings indicate that ferrichrome has the potential too be used as a first-line esophageal cancer therapy because of its low cytotoxicity and superior anti-tumor effects as compared with classical anticancer drugs, such as 5-FU. However, there are limitations to applying ferrichromes to esophageal cancer treatment based solely on this study. First, the mechanism by which ferrichrome induces DDIT3 remains unidentified. Comprehensive molecular docking simulations have indicated that ferrichrome can form various conformations and bind to multiple molecules [15]. However, it is still unclear through which of these molecules the anti-tumor effect is exerted. Additionally, because the docking simulation involved proteins with known 3D structures available in the Protein Data Bank, ferrichrome may exert its anti-tumor effect via unknown functional molecules. To identify the ferrichrome-targeting molecules, further research, such as pull-down experiments with ferrichrome added to the cancer cells and the elucidation of direct binding molecules through a mass spectrometry analysis, will be necessary. Next, while the safety of ferrichrome was evaluated by monitoring the weight changes and performing blood tests in mice, the differences in drug reactions between humans and mice [34] suggest the potential of ferrichrome to cause toxicity in humans. Moreover, the drug delivery system of ferrichrome remains unknown. To address these issues, it is essential to conduct toxicity studies using preclinical models, such as dogs and monkeys, and perform pharmacokinetic/pharmacodynamic studies of ferrichrome for a more detailed understanding of its efficacy and safety.

In conclusion, our study provides compelling evidence that ferrichrome exerts potent anti-tumor effects by inducing apoptosis through the DDIT3 pathway in esophageal cancer cells. Importantly, our safety assessments in mice have demonstrated that ferrichrome is markedly safer as compared to classical anti-tumor agents, such as 5-FU, even when administered to aged subjects. Thus, ferrichrome holds great promise as an anti-tumor agent for gastrointestinal cancers, including esophageal cancer.

## Funding statement

This study was supported by 10.13039/501100001691Grants-in-Aid for Scientific Research [Nos. 21K07929 (M. Fujiya), 22K15363 (H. Konishi), 21KK0291 (H. Konishi), and 22K08047 (K. Moriichi)], and Intractable Disease Health and Labour Sciences Research Grants from the 10.13039/501100003478Ministry of Health, Labour and Welfare, Japan(M. Fujiya).

## Ethics statement

The study received ethical approval for the use of an opt-out methodology from the Medical Ethics Committee of Asahikawa Medical University (Approval No. 16069, 20,057). All methods were performed in accordance with relevant guidelines and regulations and reported in compliance with the ARRIVE guidelines (https://arriveguidelines.org).

## Data availability statement

The datasets used and/or analyzed in this study are available from the corresponding author on reasonable request.

## CRediT authorship contribution statement

**Takehito Kunogi:** Writing – review & editing. **Hiroaki Konishi:** Validation, Supervision, Investigation. **Aki Sakatani:** Writing – review & editing, Data curation. **Kentaro Moriichi:** Supervision. **Chikage Yamamura:** Supervision, Data curation. **Koji Yamamoto:** Supervision. **Shin Kashima:** Supervision. **Katsuyoshi Ando:** Supervision. **Nobuhiro Ueno:** Supervision. **Hiroki Tanaka:** Supervision. **Toshikatsu Okumura:** Supervision. **Mikihiro Fujiya:** Supervision, Conceptualization.

## Declaration of competing interest

The authors declare the following financial interests/personal relationships which may be considered as potential competing interests:Mikihiro Fujiya and Hiroaki Konishi have received financial support from Kamui Pharma Inc. The other authors declare that they have no known competing financial interests or personal relationships that could have appeared to influence the work reported in this paper.
