# Supplementary figures and images for: Probiotic-derived ferrichrome induces DDIT3-mediated antitumor effects in esophageal cancer cells
Source: Heliyon. 2024 Mar 15;10(6):e28070. doi: 10.1016/j.heliyon.2024.e28070 (PMC10966684; doi:10.1016/j.heliyon.2024.e28070)

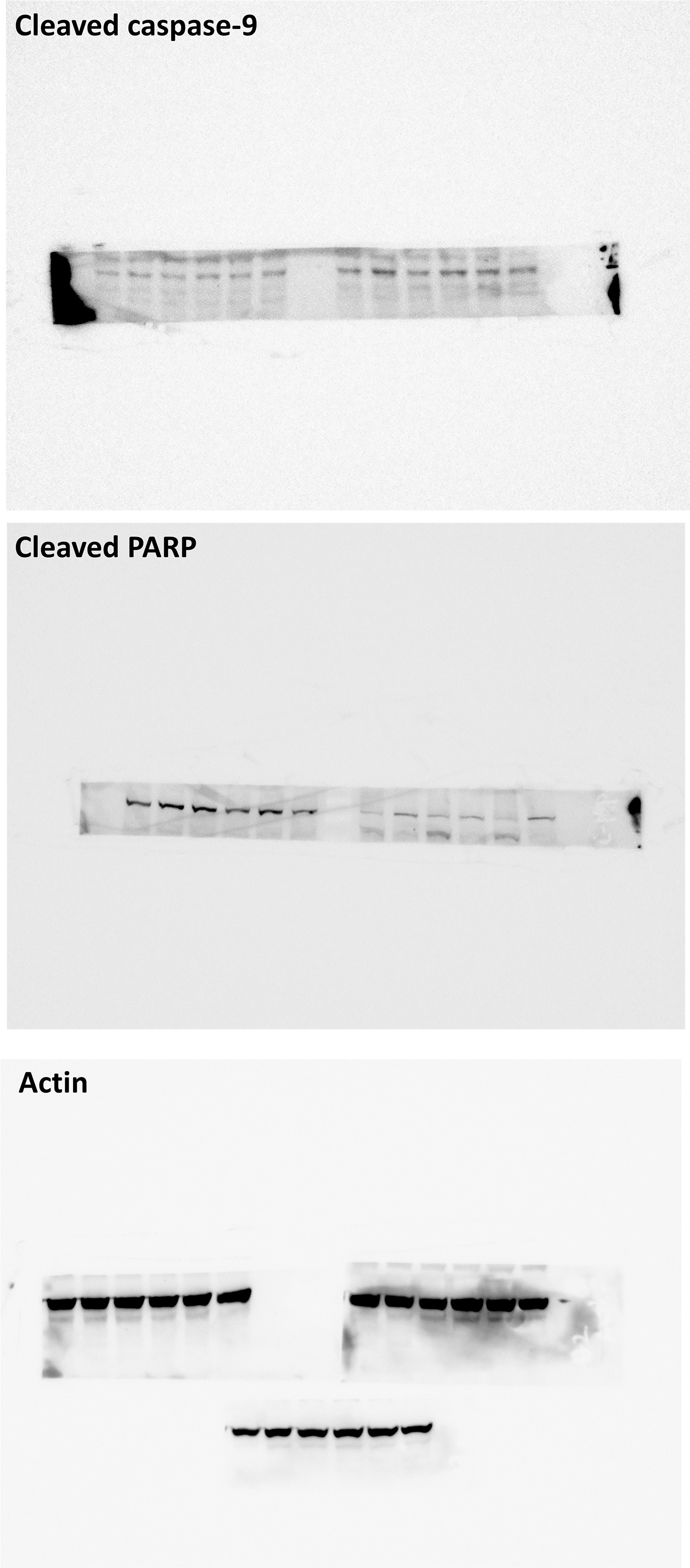

Supplement: Supplementary file 1 [file mmcfigs1.jpg]
